# Analysis of the transcriptome of bovine endometrial cells isolated by laser micro-dissection (2): impacts of post-partum negative energy balance on stromal, glandular and luminal epithelial cells

**DOI:** 10.1186/s12864-021-07713-z

**Published:** 2021-06-18

**Authors:** Wiruntita Chankeaw, Sandra Lignier, Christophe Richard, Theodoros Ntallaris, Mariam Raliou, Yongzhi Guo, Damien Plassard, Claudia Bevilacqua, Olivier Sandra, Göran Andersson, Patrice Humblot, Gilles Charpigny

**Affiliations:** 1 Department of Clinical Sciences, Swedish University of Agricultural Sciences, SLU, PO Box 7054, 750 07 Uppsala, Sweden; 2 Faculty of Veterinary Science, Rajamangala University of Technolgy Srivijaya (RUTS), Thungyai, Nakhon si thammarat 80240 Thailand; 3 Université Paris-Saclay, UVSQ, INRAE, BREED, 78350 Jouy-en-Josas, France; 4 GenomEast Platform CERBM GIE, IGBMC, 67404 Illkirch Cedex, France; 5 Université Paris-Saclay, INRAE, AgroParisTech, GABI, 78350 Jouy en Josas, France; 6 Department of Animal Breeding and Genetics, Swedish University of Agricultural Sciences, SLU, PO Box 7023, 750 07 Uppsala, Sweden

**Keywords:** Negative energy balance, Endometrium, Cell type-specific, Transcriptome, LCM, RNA-seq, Inflammation

## Abstract

**Background:**

In post-partum dairy cows, the energy needs to satisfy high milk production induces a status of more or less pronounced Negative Energy Balance (NEB). NEB associated with fat mobilization impairs reproductive function. In a companion paper, we described constitutive gene expression in the three main endometrial cell types (stromal, glandular and luminal epithelial cells) isolated by laser capture micro-dissection (LCM) showing the specificities of their transcriptomic profiles. This study investigates the specific impact of NEB on gene expression in these cells around 80 days after parturition at day 15 of the oestrus cycle and describes their specific response to NEB.

**Results:**

Following the description of their constitutive expression, the transcriptome profiles obtained by RNA sequencing of the three cells types revealed that differences related to the severity of NEB altered mainly specific patterns of expression related to individual cell types. Number of differentially expressed genes between severe NEB (SNEB) and mild NEB (MNEB) cows was higher in ST than in LE and GE, respectively. SNEB was associated with differential expression of genes coding for proteins involved in metabolic processes and embryo-maternal interactions in ST. Under-expression of genes encoding proteins with functions related to cell structure was found in GE whereas genes encoding proteins participating in pro-inflammatory pathways were over-expressed. Genes associated to adaptive immunity were under-expressed in LE.

**Conclusion:**

The severity of NEB after calving is associated with changes in gene expression around 80 days after parturition corresponding to the time of breeding. Specific alterations in GEs are associated with activation of pro-inflammatory mechanisms. Concomitantly, changes in the expression of genes encoding proteins involved in cell interactions and maternal recognition of pregnancy takes place in ST. The combination of these effects possibly altering the uterine environment and embryo maternal interactions may negatively influence the establishment of pregnancy.

**Supplementary Information:**

The online version contains supplementary material available at 10.1186/s12864-021-07713-z.

## Background

The existence of common genetic and epigenetic factors that influence metabolic imbalance, milk production and reproductive performance have been raised for long [1] and are still an important topic in dairy cow industry [2]. A significant decrease in fertility due to genetic improvement for increasing milk production has been reported for decades in dairy cows [3, 4]. Despite that a more balanced selection is currently applied [5], high milk-yield cows still meet strong negative energy balance (NEB) during the early postpartum period due to the high nutrient and energy demand for body metabolism, milk production, and body weight maintenance [6]. Energy deficiency and excessive lipid mobilization during the postpartum period have been reported to be the cause of unfavorable reproductive performances such as delayed ovarian activity [7], prolonged uterine involution period [8], retained placenta [9], endometritis [10], increased early embryonic losses and decreased conception rates [11].

Previous in vivo studies also showed the impacts of metabolic imbalance on gene expression in the endometrium during the early postpartum period [12, 13]. Other studies have reported the effect of metabolic status on endometrial transcriptome of lactating cows during the time of recognition of pregnancy [14–16]. The results suggest that NEB associated with elevated concentrations of non-esterified fatty acids (NEFAs) induces infertility in postpartum cows through dysregulation of immune pathways [12]. This is consistent with the results of in vitro studies showing that NEFAs stimulate pro-inflammatory cytokine production and lipid accumulation of endometrial cells [17] and oviductal epithelial cells [18]. However, on the one hand, the information from these in vitro models, while obtained from a single cell type and not taking into account possible interactions between the different endometrial cell types may be too simplistic and need to be confirmed in vivo. On the other hand, the previous in vivo studies regarding the impact of NEB on uterine function and endometrial transcriptome were based on RNA prepared from biopsies taken from whole endometrial tissue sections without discriminating between different cell types. Thus, the understanding of molecular changes induced by NEB from entire endometrial tissues is still unclear and difficult to interpret functionally as responses may be affected by other cell types such as endothelial cells, smooth muscle cells and leukocytes [19].

The uterus is the site of intensive tissue remodeling during the estrous cycle, at time of implantation and placental development in response to the developing embryo [20]. Reciprocally, the control of the endometrium on embryo development steps has been recently documented in mice [21]. In the cow, histology of the endometrium shows a complex association of heterogeneous structures mainly consisting of luminal epithelial cells (LE), glandular epithelial cells (GE) as well as fibroblast-like stromal cells (ST) found in different proportions in caruncular and intercaruncular tissues [22]. These three cell types are functionally responsible for the embryo implantation process under the control of steroid hormones and act in different ways [23]. For instance, bovine uterine ST synthesize and release prostaglandin E-2 (PGE-2), involved in maternal recognition of pregnancy, whereas epithelial cells contribute less to such changes in prostaglandin levels [24]. Uterine epithelial cells play key roles for the establishment and maintenance of pregnancy through activation of the innate immune system and secretion of chemokines [25] that support the recruitment and activation of immune cells directed against pathogens. Moreover, LE and GE exhibit unique molecular signatures having cooperative roles at time of establishment of pregnancy [22, 26, 27]. Their morphology [28] and biochemical activity [29] differs at time of implantation. RNA-sequencing of the complete transcriptome for the three cell types has been described for equine cells [30]. Laser capture microdissection (LCM) has also been successfully used to retrieve two different uterine epithelial cell types to define the transcriptome and proteomic analysis of the ovine and porcine endometrium, respectively [31, 32]. In the accompanying paper we have demonstrated the importance of the differences in constitutive gene expression of the different endometrial cell types isolated from biopsies performed at day-15 of the estrous cycle [33]. In this second study we report results obtained from the same biological material showing the impacts of metabolic imbalance on the response of individual endometrial cell types at time of conception, which, to our knowledge has not previously been reported. We illustrate here differences in the transcriptomic profiles, obtained by RNAseq, of luminal epithelial cells, glandular epithelial cells and stromal cells, which were harvested by LCM between cows diagnosed with either MNEB or SNEB. The changes in gene expression observed between the two types of cows, suggest the existence of long-term impacts of NEB, which appear to be mostly cell type-specific.

## Results

### Feed intake, body condition score (BCS), plasma NEFA concentrations and milk progesterone concentration

**Fig. 1:**
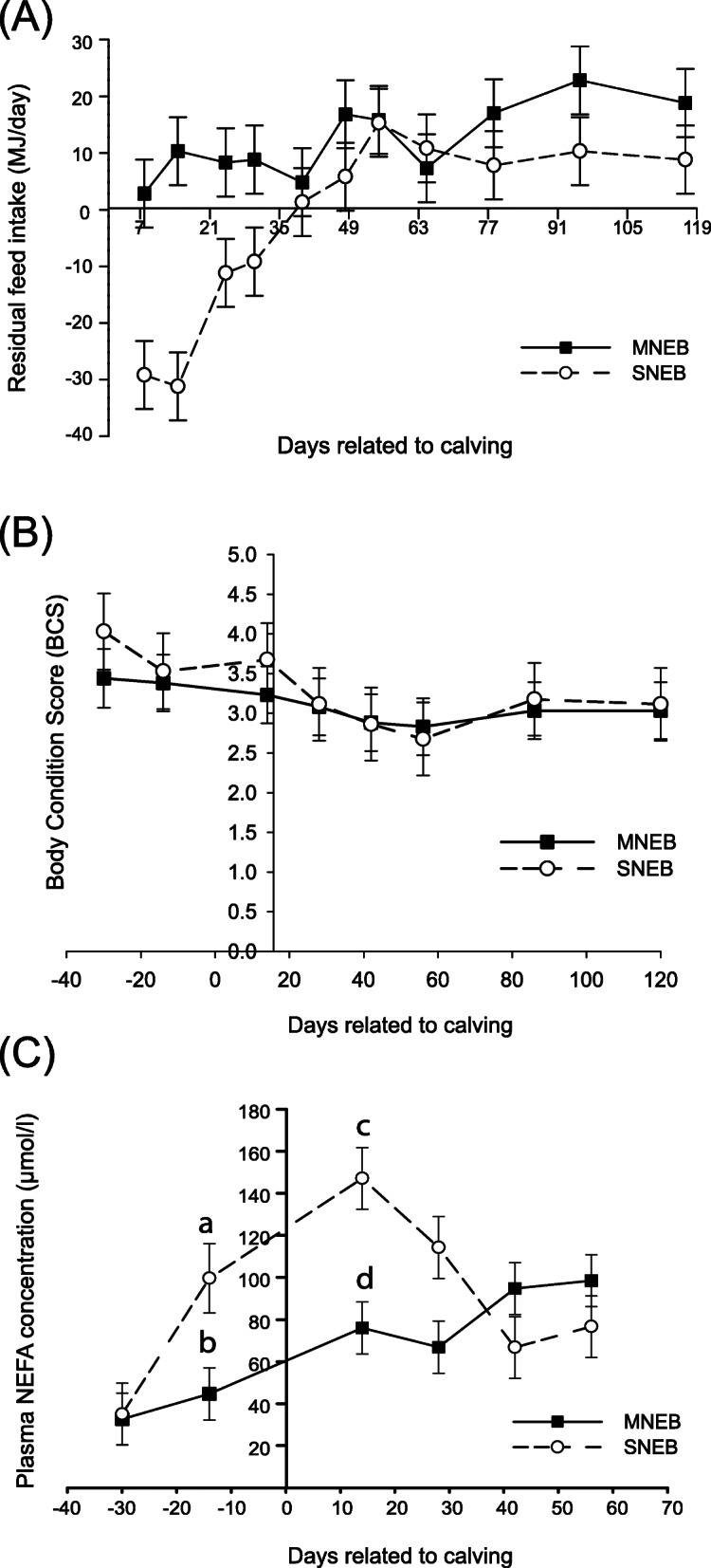
Residual feed intake (**A**), Body Condition Score (**B**) and plasma NEFA concentrations (μmol/l; LSmeans ± s.e.m.) (**C**) of LCM-selected SRB cows between observed start of the experiment and 56 days after calving in MNEB (■ solid line; *n* = 5) and SNEB (○ dashed line; *n* = 4) group. Significant differences were observed at 15 days before (a vs b; *p* < 0.05), and 15 days after calving (c vs d; *p* < 0.05)

Twelve cows in their second lactation were fed either a high or low energy diet from 30 days pre-partum. Energy balance calculations were used to classify cows as being in mild (*n* = 5) or severe (*n* = 4) negative energy balance groups (MNEB and SNEB). All cows were cyclic (one full cycle or more) before initiation of the synchronization treatment. Commencement of luteal activity was determined as day of first progesterone value above threshold of 3 ng/ml were not different (22 ± 5.6 and 23.8 ± 15.6 days in the MNEB and SNEB groups, respectively, mean ± SD). Concentrations at time or 1 day before or after biopsy at day-15 were not different in the two groups and all cows were in luteal phase at this time. The evolution of residual feed intake with post-partum time in the two groups of cows is presented in (Fig. 1A). Throughout the full experimental period, the BCS of SRB cows in both NEB groups tended to decrease (*p* = 0.08). Mean BCS was 3.65 ± 0.25 at start of the experiment and 3.05 ± 0.22 at 120 days postpartum (Fig. 1B). However, NEB did not have a significant effect on BCS. Plasma NEFA concentrations did not differ between NEB groups over the full experimental period. However, SNEB cows presented higher NEFA plasma concentrations compared to MNEB cows at Day 15 pre-partum and Day 15 post-partum (*p* < 0.05) (Fig. 1C). Reduced BCS from 30 days pre-calving and 60 days post-calving was associated with the energy balance nadir (*r* = − 0.68, *p* < 0.05). NEFA concentrations tended to be significantly associated with the residual feed intake values (*r* = − 0.28, *p* = 0.06).

### Differential gene expression between the three endometrial cell types in NEB cows

**Fig. 2:**
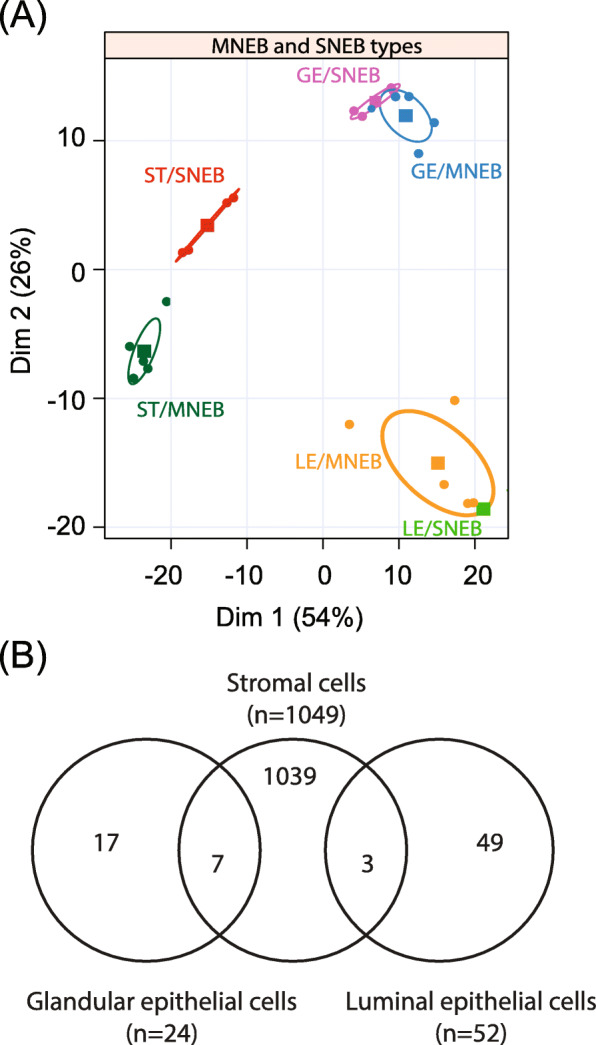
Effect of energy balance on transcriptome of endometrial cell types. (**A**) Principal component analysis of all three cell types: stromal cells (ST), glandular epithelium (GE), and luminal epithelium (LE) among two groups of cow (severe negative energy balance; SNEB and moderate negative energy balance; MNEB). (**B**) Venn diagrams from differentially expressed genes differentially expressed (DEGs) between SNEB and MNEB in each endometrial cell types (ST, GE and LE)

Endometrial biopsy samples used for analysis were collected at around 80 days post-partum on day 15 of a synchronized estrus cycle. The three endometrial cell types: luminal (LE), glandular (GE) epithelium and stromal cells (ST) were collected by laser microdissection from endometrial biopsies and the transcriptome profiles were obtained by RNA sequencing of the three cells types. Principal component analysis revealed differences in gene expression patterns in MNEB and SNEB cows for the three cell types (Fig. 2A). A clear separation between samples issued from the two groups of cows was observed in ST, whereas overlapping gene expression patterns appeared in GE and LE. The numbers of differentially expressed genes between MNEB and SNEB cows for each endometrial cell type are given in Table 1 and in the Venn diagram (Fig. 2B). The total number of differentially expressed genes (DEGs) in ST, GE and LE when comparing SNEB cows to MNEB cows were 1049, 24 and 52, respectively.

**Table 1:** Number of DEGs, which were identified as being over- or under-expressed, presented in specific endometrial cell types (ST, GE and LE) of SNEB cows when compared to MNEB cows

Expression	Cell types		
	ST	GE	LE
Over	751	15	1
Under	298	9	51
Total	1049	24	52

**Fig. 3:**
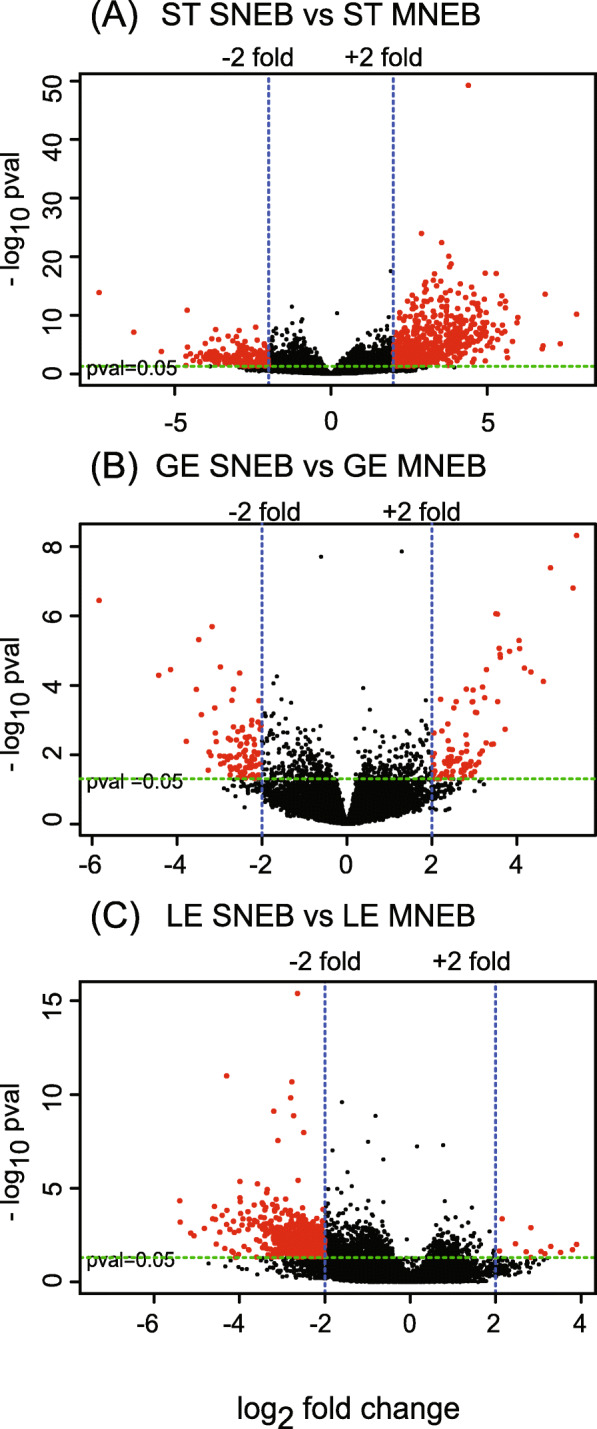
Volcano plots of distribution of differentially expressed genes between SNEB and MNEB for the three endometrial cell types ST (**A**), GE (**B**) and LE (**C**). The dotted lines in green and blue represent the cut-off, respectively for the statistical significance [−Log10 (*P*-value), y-axis] and for +/− 2 log2fold change of gene expression [x-axis]. Differentially expressed genes are shown in red dots

Seven DEGs were found as being common in ST and GE: BTG Anti-Proliferation Factor 2 (*BTG2*), Lymphocyte Antigen 6 Family Member G6C (LY6G6C), C-C Motif Chemokine Ligand 4 (*CCL4*) and JunB Proto-Oncogene, AP-1 Transcription Factor Subunit (*JUNB*), chemokine (C-C motif) ligand 3 (*CCL3*), chromobox protein homolog 1 and one pseudogene (ENSBTAG00000047824). Three DEGs were common between ST and LE: CRK Proto-Oncogene, Adaptor Protein (*CRK*), Plexin Domain Containing 1 (PLXDC) and Myotubularin related protein 10 (*MTMR10*). None of the DEGs were common to all three cell types. The list of over- and under-expressed mRNAs in ST, GE and LE are given in separate sheets of the additional file (TableS1_ DEG-SNEBvsMNEB.xlsx). In SNEB animals, a large proportion of DEGs were identified as over-expressed in ST (72%) and GE (63%) whereas almost all DEGs were under-expressed in LE (98%) (Table 1). An overview of the differential patterns of gene expression in ST, GE, and LE obtained by LCM between SNEB and MNEB cows are illustrated in volcano plots (Fig. 3A to C).

### Under-expressed genes in ST

Either by using the statistical over-representation test from PANTHER with reactome pathways annotation or by browsing pathways ontology classification, the analysis detected four main significant pathways from the 298 under-expressed genes (Table 2). A first group of genes encode proteins that are involved in the regulation of interferon signaling as well as in inflammation mediated by chemokine and cytokine (P00031). A second important group of under-expressed genes code for proteins with functions associated with the extracellular matrix and its degradation. A third group of genes code for proteins related to Wnt signaling pathway (P00057). In addition, genes of integrin signaling pathway (P00034) are over-represented. Around 10% of under-expressed genes in ST from SNEB animals are genes involved in signal transduction (GO: 0007165) and cellular response to stimulus (GO: 0051716).

**Table 2:** Gene Functional Classification Result (PANTHER 14.1) of under-expressed genes in ST cells from SNEB animals. Main pathways and ontology annotation groups enriched are shown (over-representation statistical test)

PANTHER Classification	fold Enrichment	FDR	genes
**PANTHER Reactome Pathways**			
Antiviral mechanism by IFN-stimulated genes (R-BTA-1169410)	24.99	4.03E-05	*MX2 PTPN2, JAK1, DDX58, EIF2AK2, IFIT1, ISG15, STAT1*
Interferon Signaling (R-BTA-913531)	14.9	9.55E-05	
**PANTHER GO-Slim Biological Process**			
defense response to virus (GO:0051607)	19.04	3.92E-03	*MX2, OAS1Y, IFIT1, OAS1Z, MX2, MX1*
Wnt signaling pathway (GO:0016055)	6.07	1.55E-02	*SFRP4, APCDD1, LEF1, TLE4, SULF1, WNT6, DKK3, NID1*
**PANTHER Pathways**			
Regulation of IFNG signaling (R-BTA-877312)	–	–	*RAPGEF1, MX1, EIF2AK2, UBA7, ISG15, PTPN2, MX2, DDX58, IL1RAP, IL16, CRK, IFIT1, STAT1, IFNGR2, JAK1, STX3, NFATC1, ALOX12*
Cytokine Signaling in Immune system (R-BTA-1280215)	–	–	
Antiviral mechanism by IFN-stimulated genes (R-BTA-1169410)	–	–	
Extracellular matrix organisation (R-BTA-1474244)	–	–	*KLK1, TPSB1, COL4A4, COL2A1, MMP19, NID1, COL6A6, COL4A3, COL26A1*
Collagen chain trimerization (R-BTA-8948216)	–	–	
Wnt signaling pathway (P00057)	–	–	*CDH11, TLE4, LEF1, NFATC1, PRKCH, SMARCD2, FBXW7)*
Integrin signaling pathway (P00034)	–	–	*ITGA5, ITGA10, RAPGEF1, MAP 3 K5,CRK*
Cadherin signaling pathway (P00012)	–	–	*CDH11, LEF1, CDH12, CDH2, WNT6, WNT4*
Apoptosis signaling pathway (P00006)	–	–	*EIF2AK2, RIPK1, PRKCH, MAP 3 K5*

### Over-expressed genes in ST

The GO molecular function annotation analysis using the PANTHER database (Table 3) indicates that 50% of the over-expressed genes from SNEB ST samples are distributed in three main categories: binding (GO:0005488) (*n* = 186), catalytic activity (GO: 0003824) (*n* = 130) and transporter activity (GO:0005215) (*n* = 52). Binding categories includes cytoskeletal protein binding (GO: 0008092) (*n* = 17), enzyme binding (GO: 0019899) (*n* = 24) and signaling receptor binding (GO: 0005102) (*n* = 21). Catalytic activity class includes genes involved in hydrolase activity (GO: 0016787) (*n* = 57) and transferase activity (GO: 0016740) (*n* = 47). In the transporter activity category 92% of genes are related to transmembrane transporter activity (GO: 0022857) and 8% to lipid transporter activity (GO: 0005319). Considering the PANTHER classification based on biological process annotation, the most frequently reported GO terms are cellular process (GO: 0009987; *n* = 230), cell proliferation (GO: 0008283; *n* = 105), metabolic process (GO: 0008152; *n* = 101) and localization (GO: 0051179; *n* = 72).

**Table 3:** Gene Functional Classification Result (PANTHER 14.1) of over-expressed genes in ST cells from SNEB animals. Main pathways and ontology annotation groups enriched are shown (over-representation statistical test)

PANTHER Classification	fold Enrichment	FDR	genes
**PANTHER Pathways**			
Inflammation mediated by chemokine and cytokine signaling pathway (P00031)	2.15	5.26E-03	*ACTA2, JUNB, CAMK2B, CCL11, MYH11, ACTG2,, ITPR2, PRKCZ, MYH14, ACTA1, PLCB4, MYLK, PAK4, PLCH1, CCL4, CCL3*
Wnt signaling pathway (P00057)	2.1	2.58E-02	*ACTG2, FZD5, PLCB4, CDH3, PRKCZ, CDH1, ACTA1, CTBP2, ITPR2, FRZB, ANKRD6, ACTA2*
Cadherin signaling pathway (P00012)	2.21	3.43E-02	*ACTA2, CDH3, ACTG2, FRK, FZD5, ACTA1, CDH1, ERBB3*
Integrin signaling pathway (P00034)	1.72	1.51E-02	*ACTG2, ITGB4, FRK, RAP2A, ITGB6, FLNA, COL4A6, ACTA1, FLNB, COL4A5, ACTA2*
Gap junction trafficking and regulation (R-BTA-157858)	4.98	1.16E-02	*GJB4, GJB1, GJB3, GJB5*
Cytoskeletal regulation by Rho GTPase (P00016)	3	7.29E-03	*ACTA2, MYH11, ACTG2, ARHGAP1, MYH14, ACTA1, MYLK, PAK4*
**PANTHER Biological Process**			
cytoskeleton-dependent intracellular transport (GO:0030705)	3.49	6.77E-03	*RP1, ACTA2, SPAG17, ACTG2, BICDL2, JHY, JHY, ACTA1, TEKT4, CENPJ, DYNC1I1, CFAP54, CCDC39, DRC1, BICDL1*
cilium movement (GO:0003341)	7.88	7.77E-03	*DNAH7, ZBBX, TACR1, TEKT4, CCDC39, DNAH11, DRC1*
**PANTHER Molecular Function**			
transmembrane transporter activity (GO:0022857)	1.55	1.54E-02	*LRRC26, SLC45A2, P2RX3, LRRC38, KCNG1, SLC36A2, SLC38A11, KCNMB1, CLCN3, GRIN1, ATP1B1, ATP2B4, SLC18A2, SLC5A9, SLC29A2, CACNB2, GRIA3, KCNF1, SLC5A10, SGK2, LRRIQ1, ATP6V1G3, SLC35A3, SLC4A7, SLC10A1, KCNH2, TMC5, SLC31A2, SLC34A2*

The analysis from PANTHER pathways (Table 3) revealed that genes from three significant pathways are over-represented in ST from SNEB vs MNEB cows including: (i) genes related to inflammation mediated by chemokine and cytokine signaling pathway (P00031); ii) genes involved in Wnt signaling pathway (P00057) and in cadherin signaling pathway (P00012); and (iii) genes associated to integrin signaling pathway (P00034). In addition, according to Biological Process classification, a positive enrichment was detected for genes related to cytoskeleton-dependent intracellular transport (GO:0030705) and for cilium movement and dynein complex (GO:0003341). Finally, many genes involved in transport activity highlight the enrichment of transmembrane transporter activity (GO:0022857).

### Differential expression in GE

Only nine known genes are under-expressed in GE cells from SNEB cows when compared to MNEB ones (*NPPB, PTGFR, ANKS1B, CDH18, PPP1R1C, LY6G6C, MT1E, ASB16* and *PROM2*). Five are related to binding functions (*PROM2, CDH18, NPPB, PTGFR* and *MT1E*) and/or involved in biological regulation (*CDH18, NPPB, PTGFR* and *MT1E)*. Due to the limited number of under-expressed genes, no enrichment could be detected in statistical overrepresentation test. Among the 15 over-expressed genes, three main pathways are over-represented (Table 4). Four genes (*JUNB, CCL2, CCL4* and *CCL3*) encode proteins with functions related to inflammation mediated by chemokine and cytokine signaling pathway (P00031 and GO:0070098). Four genes encoding immediate-early transcription factors (*FOS, JUNB ATF3* and *EGR2)* are associated with numerous related annotation terms: RNA polymerase II proximal promoter sequence-specific DNA binding (GO: 0000978), DNA-binding transcription factor activity, RNA polymerase II-specific (GO:0000981). An additional enriched pathway is revealed (Gonadotropin-releasing hormone receptor pathway; P06664).

**Table 4:** Gene Functional Classification Result (PANTHER 14.1) of over-expressed genes in GE cells from SNEB animals. Main pathways and ontology annotation groups enriched are shown (over-representation statistical test)

PANTHER Classification	fold Enrichment	FDR	genes
**PANTHER Pathways**			
Inflammation mediated by chemokine and cytokine signaling pathway (P00031)	24.95	9.01E-04	*JUNB, CCL2, CCL4, CCL3*
Gonadotropin-releasing hormone receptor pathway (P06664)	31.19	3.17E-05	*JUNB, FOS, ATF3, NR4A1*
Apoptosis signaling pathway (P00006)	37.42	2.83E-03	*FOS, ATF3*
**PANTHER GO-Slim Biological Process**			
chemokine-mediated signaling pathway (GO:0070098)	> 100	5.44E-04	*JUNB, CCL2, CCL4, CCL3*
**PANTHER Molecular Function**			
DNA-binding transcription factor activity, RNA polymerase II-specific (GO:0000981)	21.8	2.30E-04	*JUNB, FOS, ATF3, EGR2*
cytokine receptor binding (GO:0005126)	36.04	1.75E-03	*CCL2, CCL4, CCL3*

### Differential expression in LE

In LE samples, only *B4GALT5* is over-expressed in SNEB. No significant enriched GO terms is related to the under-expressed DEGs at FDR *p* value < 0.05. By using the raw P value instead FDR (Table 5), the analysis of under-expressed genes indicates some enrichments corresponding to signaling receptor binding (GO:0005102), cytokine activity (GO:0005125) and steroid binding (GO:0005496). Other genes belonging to relevant pathways are highlighted by scanning the list of DEGs: defense response to bacterium (GO:0042742); signaling by PDGF (R-BTA-186797); Synthesis of Leukotrienes and Eoxins (R-BTA-2142691).

**Table 5:** Gene Functional Classification Result (PANTHER 14.1) of over-expressed genes in LE cells from SNEB animals. Main pathways and ontology annotation groups enriched are shown (over-representation statistical test)

PANTHER Classification	fold Enrichment	raw ***P*** value	genes
**PANTHER GO-Slim Molecular Function**			
signaling receptor binding (GO:0005102)	4.21	6.60E-03	*BMP2, SEMA6D, IGHV2, NDP, TAP*
cytokine activity (GO:0005125)	7.42	3.05E-02	*BMP2, NDP*
steroid binding (GO:0005496)	25.23	3.13E-03	*PAQR8, OSBPL6*
**PANTHER GO-Slim Biological Process**			
response to steroid hormone (GO:0048545)	37.97	2.79E-02	*PAQR8*
defense response to bacterium (GO:0042742)	10.07	1.75E-02	*IGHV2, TAP*
**PANTHER pathways**			
Signaling by PDGF (R-BTA-186797)	44.52	5.36E-05	*PTPN12, THBS4, CRK*
Synthesis of Leukotrienes (LT) and Eoxins (EX) (R-BTA-2142691)	33.64	3.12E-02	*CYP4B1*

### KEGG pathway analysis of the DEGs

Significantly enriched KEGG pathways from DAVID database were found in GE and ST, whereas no significant KEGG pathway was detected in LE (Table 6). In ST cells, DEGs between SNEB and MNEB cows were significantly enriched in four different KEGG pathways. Furthermore, the David database recognized 25 different KEGG pathways with the overexpressed genes. Two were found significantly enriched. They are related to calcium signaling pathway (KEGG map 04020, fold enrichment = 3.4; 17 DEGs) and tight junctions (KEGG map 04530; fold enrichment = 4.8; 11 DEGs. With under-expressed DEGs, two KEGG pathways associated with viral infectious diseases (KEGG “measles” map 05162 and KEGG “Influenza A” map 05164; fold enrichment respectively = 5.0 and 4.1; 11 DEGs) are overrepresented (Table 6). The names of these two KEGG pathways do not make sense with endometrial physiology. The genes of these pathways are known to be important members of interferon signaling that is a critical mechanism for establishment of pregnancy (reactome pathways: BTA-913531, BTA-877312). For glandular epithelium, over-expressed DEGs matched to 10 overrepresented KEGG pathways. The KEGG TNF signaling pathway (KEGG map 04010) was the only one found to be significantly enriched (Fold enrichment = 21.5). In contrast, no enriched KEGG pathways were found from the set of under-expressed DEGs.

**Table 6:** The significant KEGG pathways with over- or under-expressed DEGs for three endometrial cell types (ST, GE and LE) were identified using DAVID database (adjusted *p*-value < 0.05)

cell type	under/over	KEGG Pathway Id	pathway name	genes
Stromal cells	over-expressed	map 04020	calcium signaling	*P2RX3, ITPKA, ITPR2, CHRM3, ERBB3, CAMK2B, PLN, PLCB4, SLC25A4, HTR2A, MYLK, ADCY8, TACR1, PTGFR, PTGER3, RYR3, GRIN1*
		map 04530	tight junction	*OCLN, IGSF5, MYH14, CLDN23, PRKCZ, MYH11, CGN, TJP3, LLGL2, CLDN8, CLDN3*
	under-expressed	map 05162 map 05164	measles and influenza A	*JAK1, DDX58, ADAR, STAT1, IFIH1, EIF2AK2, IFNGR2, MX1, OAS1Z, OAS1Y, IRF7*
Glandular cells	over-expressed	map 04010	TNF signaling	*FOS, SOCS3, JUNB, CCL2*

**Fig. 4:**
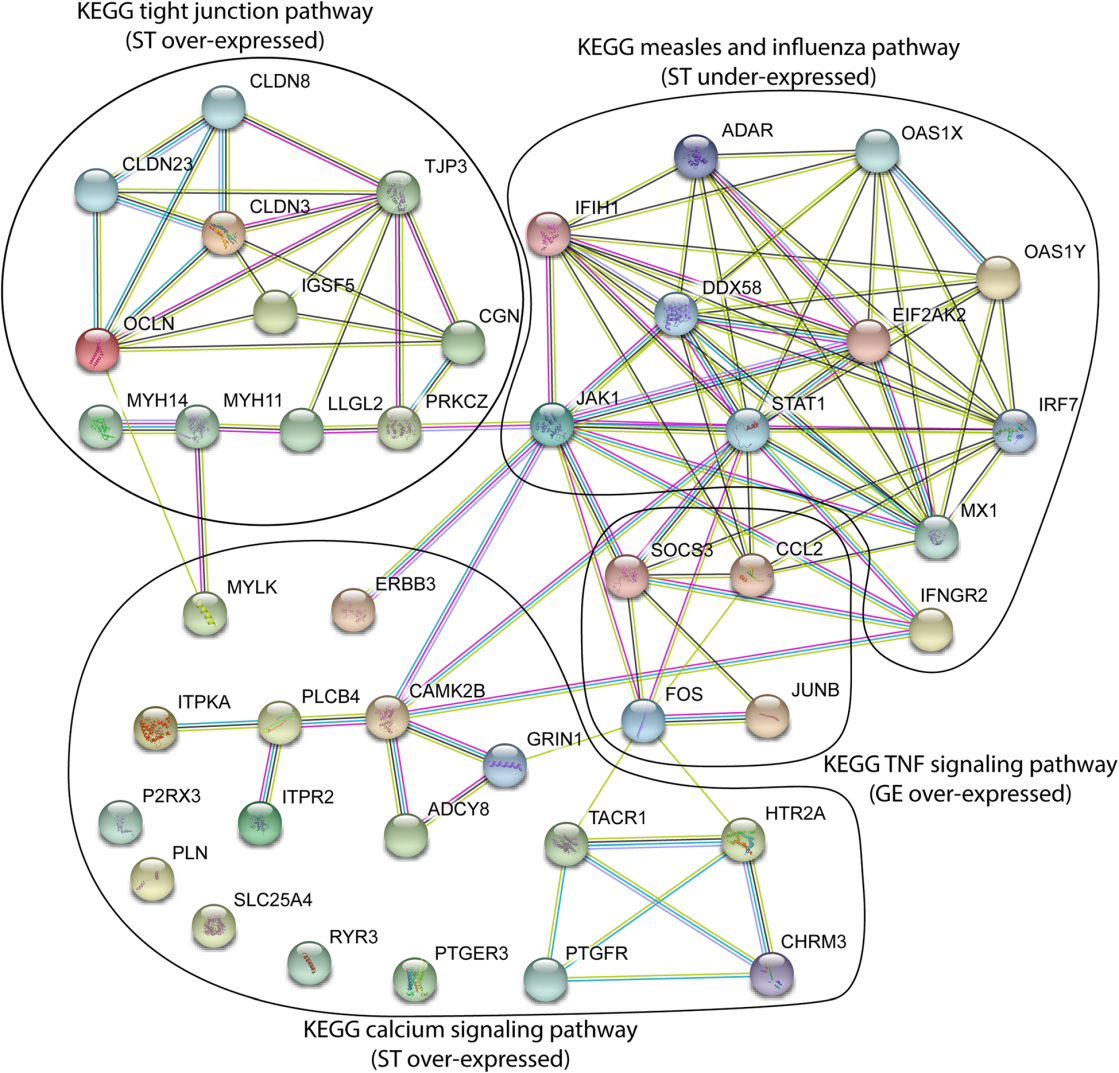
Protein association network visualization in STRING. DEGS of ST and GE endometrial cell types selected from significant KEGG pathways (Table 6). Four functional modules can be seen in the network forming tightly connected clusters centered on JAK1 and STAT1 proteins. Two functional modules are related to ST over-expressed genes in SNEB (respectively tight junction pathway and calcium signaling pathway). One functional module is associated to ST under-expressed genes in SNEB (measles and influenza pathway). One functional module is related to GE over-expressed genes in SNEB. Line color indicates the type of predicted associations and supporting evidence at medium confidence score (0.4) (see STRING web site for color legends)

The corresponding STRING-generated interaction network obtained from DEGs belonging to the 5 KEGG pathways associated to ST and GE cells revealed strong interactions (PPI enrichment value < 1.0E-16) between these sets of DEGs that are related to the JAK/STAT signaling (Fig. 4)*.*

## Discussion

During negative energy balance (NEB), lipolysis in adipose tissue is increased resulting in decreased BCS and increased NEFAs in blood [34]. Changes in BCS and NEFA concentrations were correlated with NEB nadir and plasma NEFA concentrations in SNEB cows were greater than in MNEB cows in the pre-partum and early post-partum. Both observations are consistent with earlier findings [35] and shows that the two groups were in a different metabolic status before and during the two first weeks post-partum. The impacts of NEB on bovine reproductive performances are well documented [36]. A wealth of information illustrates the negative effects of NEB and NEFA on ovarian cells [37], embryos [38] and oviduct [39]. On the contrary, relatively few publications have reported effects of NEB on the endometrial tissue or cells. In vivo studies showed that NEB had negative impacts on endometrial function through the alteration of immune response and activation of pro-inflammatory and IGF-insulin signaling pathways [12, 40]. However, in those studies and others [14–16] information was obtained from full tissue and to our knowledge, the present study is the first time that the specific effects of NEB on the three main cell types of the endometrium are reported.

### Impact of NEB on the three endometrial cell types

Overall, our results show that NEB impacts mainly ST whereas GE and LE cells are less affected. More than 10% (13%) of the total number of genes expressed in ST were impaired by NEB status while less than 1% were affected in GE and LE (0.3 and 0.7%, respectively). When considering the sub groups of genes showing a specific expression related to cell type, NEB did not affect any of those in GE and modified only the expression of *TCN1* and *B4GALT5* in LE cells. This number is probably under-estimated in LE due to the comparison restricted to a single sample in the SNEB group. By contrast, a relatively high number of genes (about 8%; *n* = 91) specifically expressed by ST are affected by NEB.

### Impact of NEB on genes related to cytoskeleton and cell adhesion

Genes encoding tropomyosins (*TPM1* and *TPM2*) and myosins (*MYO5C* and *MYO5B*) proteins, which are structural constituents of cytoskeleton (GO: 0016459) were over-expressed in ST of SNEB cows. Similar over-expression of tropomyosins and myosins has been reported in the endometrium of fertile cows [41]. The increased expression of myosins was associated to over-expression of genes of the dynein family (*DNAH5, DNAH7, DNAH11, DYNC1I1* and *DYNLRB2*), which encode proteins that are involved in cell mobility (GO: 0005874). The signification of these changes in the context of fertility deserves further investigations. In contrast, a large set of genes related to cell adhesion and cell-cell and cell-extracellular matrix adhesion [42], such as integrins (*ITGA5* and *ITGA10*), cadherins (*CDH2, CDH11* and *CDH12*), *AGRN*, *EGFLAM, TGFBI*, type IV collagen (*COL4A4*), type VIII collagen (*COL8A1*), *ODZ3, SCARB2* and *WISP3* were under-expressed in ST of SNEB cows. The lower expression of integrins could be seen as unfavourable to establishment of pregnancy. In humans, *ITGB3* mRNA has been cited as a positive marker associated with pregnancy [43, 44]. In sheep, elevated expression of *ITGAV*, *ITGA4*, and *ITGA5* in GE has been found during pregnancy [45]. E-cadherin (*CDH1*) has been documented as a critical gene for embryo implantation as its under-expression in epithelial cells allows endometrial cells dissociation following blastocyst invasion [46]. Moreover, an increased expression of type IV collagens has been identified in endometrium of low fertility heifers [47], however, the opposite trend was found here in SNEB cows. In ST from the SNEB group, genes belonging to the Wnt pathway (P00057) were either over-expressed (*ACTG2, FZD5, PLCB4, CDH3, PRKCZ, CDH1, ACTA1, CTBP2, ITPR2, FRZB, ANKRD6* and *ACTA2)* or under-expressed (*CDH11, TLE4, LEF1, NFATC1, PRKCH, SMARCD2* and *FBXW7).* These genes encode proteins that are associated with GO: 0001763 (morphogenesis of a branching structure) GO: 0001944 (vasculature development) including involvement in the morphogenesis and function of the endometrial glands [48, 49] as well as in the development of uterine vasculature [50]. The altered expression of these genes by the NEB can have a critical role in the regeneration of the endometrium during the post-partum period.

### Impact of NEB on genes related to energy metabolism

In SNEB cows, among the 700 genes that are over-expressed in ST, a large proportion were genes classified to encode proteins related to metabolic process (GO: 0008152), macromolecule metabolic process (GO: 0043170) and organic substance metabolic process (GO: 0071704). DEGs were most particularly related to catalytic activity (GO: 0003824) revealing the breakdown of nutrient molecules to supply energy to cells. This suggest that SNEB cows still presented an energy deficit in endometrial cells at time planned for breeding, despite that energy balance is progressively restored. SNEB cows presented also an increased expression of many genes encoding proteins with functions related to lipid metabolism (fatty acids, triglyceride and cholesterol metabolic processes) such as *ACSM3, CPT1B, LPL, PPARGC1A, PRKAA2, GGT1, PLA2G10, CYP2B6, CYP2C18, HACD1, SLC27A6* and *PLIN4* in ST. Four of them *CYP2B6, CYP2C18, PLA2G10,* and *GGT1* are involved in arachidonic acid (AA) metabolism. While the release of AA following phospholipase activation is usually engaged in the production of endometrial prostaglandins via cyclooxygenases enzymes, the conversion of AA by CYP enzymes contribute to oxidative stress and inflammation and may not be favourable to endometrial function [51]. The receptivity of fibroblasts to prostaglandins could also be modified through their receptors with the observed extreme over-expression of *PTGFR* mRNA (the second top of over-expressed DEGs in ST) and *PTGER3*. The over-expression of *SLC27A6,* a gene encoding a fatty acid binding protein (FABP) [52] and *PLIN4,* which controls intra-cellular lipid droplet-associated proteins, are consistent with earlier findings in obese mice and human [53, 54]. Our data showing associations between over-expression of these genes with increased plasma NEFA concentrations are consistent with the over-expression of genes of the PLIN family found in the endometrium of low fertility heifers [47]. Taken together, this information suggests that up-regulation of genes involved in lipid uptake in ST of SNEB cows, associated with elevated NEFA concentration during the peri-parturient period may be unfavourable to fertility in post-partum cows. Increased gene expression from the solute carrier family in ST from SNEB cows (such as *SLC2A12, SLC45A2* and *SLC35A3*), which encode proteins involved in carbohydrate transportation, could be seen as a compensatory mechanism as the under-expression of the glucose transporter (*SLC2A1*) mRNA was detected in endometrial tissue of subfertile dairy cows [55].

### Impact of NEB on genes related to growth factors

Interestingly, expression of genes associated with IGF-insulin signaling, such as *IGF1R* and *IGF2BP2*, was higher in SNEB cows. On the contrary, *IGFBP2, GDF6, EDIL3* and *TGFBI* were under-expressed in ST of SNEB cows. The expression of IGFs were detected in the uterine stroma especially in the caruncular areas of cyclic cows [56]. As suggested in the above-referred study and by others [40], the dysregulation of genes related to insulin-like growth factors function may delay tissue remodelling during the post-partum period. In our study, the importance of those changes on matrix metalloproteinase (MMP) appeared limited as only one gene of the MMPs family (*MMP19*) was under-expressed in ST of SNEB cows. However, 9 closely related genes coding for proteins involved in the degradation of the cellular matrix and tissue remodelling were also under-expressed in the SNEB cows. On the contrary, growth factor receptors such as *GRB7, GRB14* and *FGFR2*, which are known as stromal-derived paracrine stimulators of epithelial proliferation, were over-expressed in ST of SNEB. This increase may be a mechanism for compensating endometrial epithelial defects in order to achieve uterine receptivity [57]. In bovine species, gene expression of FGFs and their receptors is upregulated during pregnancy and these factors stimulate interferon-tau (*IFN*-*T*) production during the pre-attachment phase of conceptus development [58]. The increase of transcripts encoding proteins of the cyclin family (*CCND3* and *CCNB1*) in ST of SNEB cows may also be associated with the modifications of proliferative properties and tissue differentiation in the endometrium for preparing embryo implantation [59]. Our results show that NEB status influences both the over-expression and under-expression of genes encoding numerous different growth factors. However, further studies are needed to decipher the consequences of these changes and how they may affect fertility.

### Impact of NEB on genes related to inflammatory responses

Nearly 20 genes belonging to two pathways [cytokine signaling in immune system pathway (R-BTA-1280215) and inflammation mediated by chemokine and cytokine signaling pathway (P00031)] displayed reduced transcripts in ST of SNEB. Among these genes *JAK1* and *STAT1* have been associated with both IFN-γ and IFNα/β endometrial receptors [60]. It may be hypothesized that the reduced-expression of *JAK1* and *STAT1* may alter JAK/STAT signaling and immune response in stromal cells. Indeed, a large number of IFN-inducible genes (R-BTA-877312), such as *MX1, MX2, IFI44, IFI6, IFIH1, IFIT1, IFITM2* and *IFNGR2* were under- expressed in ST of SNEB cows. These findings are different from previous observations showing over-expression of *MX1* and *MX2* genes in the full endometrium of SNEB cows during early postpartum [12]. The specificity of stromal cell response to SNEB, may explain differences between studies, however due to the lack of effect on GE, these discrepancies may result also from differences in time post-partum and severity of NEB. The glandular epithelium plays a major role in the activation of the innate immune system as reviewed by [61]. In our study, most of the DEGs in GE related to chemokines, immune response processes, TLRs and TNF signaling pathways, such as *CCL2, CCL3, CCL4, CCL11, FOS, JUNB,* and *SOCS3* were strongly over-expressed in SNEB cows. Some of those genes belonged to the C-C motif chemokine ligands (CCLs) family and play an important role in monocyte recruitment in the endometrium [62]. Increased expression of *CCL2* mRNA was found associated with lipid accumulation induced uterine inflammation in obese rats [63]. The present results are similar with previous studies performed with full endometrial tissue, showing the up-regulation of inflammatory response genes in SNEB cows [40]. This is also consistent with several studies in other mammals showing that metabolic imbalance, increased lipolysis and most particularly NEFAs, play essential functions in the activation of TNF and TLRs signaling to promote the release of pro-inflammatory molecules [64, 65]. Taken together, these studies and our present findings suggest that SNEB and NEFAs activate pro-inflammatory pathways in the glandular epithelium and stromal cells. On the contrary, in luminal epithelium, the adaptive immune response (B cell-mediated immunity) and innate immunity, was represented by under-expressed genes such as tracheal antimicrobial peptide (*TAP*), a beta-defensin gene, which was associated to the NF-κB pathway [66], and by genes coding for immunoglobulin heavy variable chains that participates in the antigen recognition. These observations need further confirmation. Our results indicate that SNEB induces changes in immune responses, which are different in the three endometrial cell types. They show also that these changes are still present, long after NEB has disappeared suggesting long term effects of metabolic imbalance and NEFAs on the pro-inflammatory status of the glandular epithelium and the stroma.

### Effect of NEB on genes related to maternal-conceptus recognition

A large set of IFN-inducible genes such as *MX1, MX2, STAT1, JAK1, IFIH1, IFNGR2, ISG15, LY6G6C, OAS1Y, OAS1Z* and *IRF7* were under-expressed in ST of SNEB cows. A weaker expression of those genes that encode proteins involved in IFN-T signaling could account for the decreased endometrium-related fertility in SNEB cows. In pregnant ruminants, IFN-T is the main pregnancy recognition signal [67], that allows the persistence of the corpus luteum and maintaining elevated progesterone concentrations by blocking oxytocin signaling and PGF2α secretion [68]. Oxytocin signaling has been associated with the maintenance of gap-junctions in luteal tissue [69] and intracellular calcium release in endometrial cells [70]. Differentially expressed genes and our STRING protein-protein network revealed in ST of SNEB cows showed an increase in expression of six genes encoding proteins belonging to the oxytocin signaling pathway namely *PLCB4, ADCY8, CAMK2B, ITPR2,* and *MYLK* (Fig. 4). These changes are consistent with the over-expression of 10 genes related to tight junction such as *MYH14, MYH11, PRKCZ, OCLN, CGN, IGSF5, TJP3, CLDN3, CLDN8* and *CLDN23*. Over-representation of oxytocin signaling genes together with under-expression of genes involved in pregnancy maintenance suggest a potential weakness of antiluteolytic mechanisms in the SNEB group. The changes in ST are consistent with downstream changes related to PGF2α produced by both endometrial epithelial and stromal cells [71]. Furthermore the deregulation of this signaling pathway in SNEB cows is supported by changes in levels of *PTGFR* mRNA, which was over-expressed in ST but under-expressed in GE. In addition, other important genes encoding proteins with established functions critical for implantation such as *IL1RAP, SOSC3* and *AREG* were found to be differentially expressed in SNEB cows. We observed a lower expression of the *IL1RAP* gene in ST of SNEB cows. The IL1RAP protein is a necessary part of the interleukin 1 receptor complex and is regulated by interleukin 1 beta (IL-1β)-dependent signaling. The over-expression of *IL1R* and *IL1RAP* mRNAs under IL-1β regulation has been reported in the pig endometrium at day 12 of pregnancy to stimulate the expression of *PTGS1* and *PTGS2* genes which encode key enzymes for PGE2 and PGF2α synthesis [72]. Blocking IL1R signaling with an IL-1 receptor antagonist led to implantation failure in mice [73]. The reduced expression of *IL1RAP* in ST of SNEB cows may compromise the establishment of pregnancy, but this deserves further investigation in the cow. SOCS family genes (*SOCS1–7*) inhibit cytokine signaling through the JAK–STAT pathway and regulate IFNs, growth factors and hormones which are critical for implantation [74]). *SOCS1–3* mRNAs are over-expressed at time of implantation in the endometrium of pregnant cows and their expression was induced by IFN-tau in endometrial cells in vitro [75]. The over-expression of *SOCS3* mRNA in GE may contribute to down regulate the JAK/STAT signalling pathway in the neighbouring ST cells, as reported above. *AREG* was over-expressed in GE of SNEB cows. *AREG* gene is known as an epidermal growth factor receptor and is involved in cell growth, proliferation, differentiation and migration. It is highly expressed in luminal and glandular epithelium during the secretory phase of menstrual cycle and early pregnancy in human and primate [76]. As for *SOCS3,* it could be speculated that the over-expression of *AREG* mRNAs in GE may be part of a compensatory mechanisms in response to the increased expression of cytokines in these cells. It would be interesting to compare the amplitude of over-expression of *SOCS3* and *AREG* observed in the present situation (luteal phase under cyclic conditions) with levels during pregnancy to evaluate possible impacts of NEB on implantation.

## Conclusion

The present study provides novel and specific information about differential gene expression in three endometrial cell types from post-partum dairy cows. We show that the impacts of negative energy balance on the gene expression of endometrial cells are cell type-specific. Major and specific changes in gene expression were observed in stromal cells illustrating dysregulation of metabolic processes especially lipid and carbohydrate metabolism, cytoskeleton and cell adhesion properties. Altered gene expression of endometrial epithelial cells under SNEB condition was related to activation of pro-inflammatory responses via chemokine pathway in GE, whereas down-regulation on adaptive immunity and defence mechanism were found in LE. Strong changes in the expression of genes encoding proteins involved in prostaglandin production and maternal-conceptus recognition was found in ST and in GE. Considering the above and the crucial role of IFN-tau for embryo implantation and maintenance of pregnancy, our hypothesis is that the under-expression of IFN-tau responsive genes associated with the increased expression to oxytocin and PGF2α related genes may be detrimental for the establishment of pregnancy in SNEB cows. The changes in gene expression induced by NEB in LE should be considered as preliminary and needs further confirmation whereas the specific response of ST and GE to NEB paves the way for functional studies relating the importance of these changes for the establishment of pregnancy.

## Methods

### Animals and experimental design

This study was approved by the Uppsala Animal Experiment Ethics Board (application C329/12, PROLIFIC). After the study was conducted all cows have been kept in usual farm living conditions. All studies were conducted at the Swedish Livestock Research Centre in Lӧvsta, Uppsala, Sweden. The animals were kept in a loose housing barn with a voluntary milking system (VMS, DeLaval, Tumba, Sweden), and had free access to drinking water. The current paper reports the specific impacts of post-partum negative energy balance (NEB) on stromal, glandular and luminal epithelial cells whereas a companion paper based on the same biological material reported the differences of the transcriptome between cell-types [33]. Second lactation cows of the Swedish Red breed (SRB; *n* = 12) were fed two different diets i.e. i) high-energy diet (control, *n* = 6) targeting 35 kg energy-corrected milk (ECM) and ii) low-energy diet targeting (*n* = 6) 25 kg energy-corrected milk (ECM) which was achieved by giving to these cows 50% concentrate. For each cow, the differential diets were given between 30 days pre-partum and 120 days post-partum. The dietary details, management conditions and relationships with metabolic response and NEB profiles were previously described [35]. During the experiment, consumption of concentrate was individually adjusted with an automatic feeding machine while forage was fed ad libitum. All cows initially recruited in the experiment were checked for uterine health by using both clinical examination, including ultra-sound examination and endometrial cytology. All cows included in further experiments (synchronization of estrus followed by uterine biopsies in view of LCM) had no clinical signs of uterine disease [77]. They presented less than 10% (four cows had percentages of immune cells between 7 and 10%, and all other cows presented less than 5% of immune cells counted from a total of 400 cells) of immune cells from endometrial cytobrush at 42–45 days post-partum, according to [78]. At day 60 after calving, estrous was synchronized using an intra-vaginal progesterone device (CIDR, Zoetis, Parsippany, NJ, USA) for a week followed by i.m. injection of 500 μg of prostaglandin analog (Estrumate®, MSD animal health, Madison, NJ, USA) intramuscular as described [79]. Fifteen days after visual oestrus detection, endometrial tissue biopsies were collected under epidural anesthesia with 0.5 mg/kg of 1% lidocaine hydrochloride (1% Xylocaine®, Astra Zeneca, Cambridge, UK). Timeline for sampling and analysis of phenotypic responses are presented in supplemental Figure S1. Biopsies were collected at day 15 of the cycle, i.e. at a time progesterone, concentrations which modulate temporal changes in endometrial gene expression are high (reviewed by Forde et al. [23]. This limits also possible individual differences in the dynamics of progesterone patterns associated with luteolysis, which may occur later on.

### Energy balance (EB) calculation and classification

The energy balance (EB) (residual feed intake (RFI) expressed in MJ/day) was calculated as the difference between energy consumed and energy used for milk production, body maintenance, growth and pregnancy for each individual cow. Calculations were performed once every week from first week after calving to day 120 as described in [35]. All data used were routinely recorded in the university herd and energy balance calculation was performed with NorFor used as the reference system in the Nordic countries. Based on the analysis of NEB data, 9 out of 12 cows with the most differentiated profiles were classified into two NEB groups with either a mild negative energy balance (MNEB) group (*n* = 5) or a severe negative energy balance (SNEB) group (*n* = 4). Endometrial biopsy samples from these nine cows were subsequently used for LCM and RNA sequencing. Residual feed intake values in the first week postpartum of these nine cows ranged from − 52.77 to 21.26 MJ/day and means (± s.e.m.) of 1.30 ± 6.35 and − 29.48 ± 7.10 MJ/day were observed in the MNEB and SNEB groups, respectively.

### Body condition score (BCS) and plasma NEFA measurements

Body condition score (BCS) was evaluated and recorded by the same person every 2 weeks, from 30 days pre-partum until 120 days post-partum. BCS was used on a 5 point scale with 0.5 point increments, 1 = very lean to 5 = fat [80]. Blood samples were taken every 2 weeks from the coccygeal vein in EDTA containing tubes (BD Vacutainer, Kremsmünster, Austria) from 30 days pre-partum to 56 days post-partum and then centrifuged at 4000 g for 10 min at 4 °C. Following centrifugation, plasma samples were distributed into 0.5 mL aliquots and stored in − 20 °C until NEFA analyses were performed. NEFA concentrations were measured in duplicate by using a non-esterified fatty assay kit (Bio Scientific Corporation, Austin, TX, USA) with detection range 0–4 mM. The intra- and inter-assay variability was 4.19 ± 3.99% and 2.63 ± 1.08%, respectively.

### Milk progesterone measurements and estrous cycle stage at time of biopsies

Whole milk samples were collected by the automatic milking machine, VMS (DeLaval) three times per week from Day 7 to Day 120 after calving. Milk progesterone concentrations were measured with a commercial enzyme-linked immunosorbent assay (ELISA) (Ridge way ‘M’ kit, Ridgeway Science, Gloucester, UK) as previously described [35]. The progesterone concentration profile was used to determine the estrous cycle stage at the time of biopsy sampling. All cows selected were in the luteal phase at time of endometrial biopsy as shown by their mean (± s.e.m.) progesterone concentration (8.78 ± 2.12 ng/mL; range from 6.66 to 10.90 ng/ml).

### Collection of endometrial biopsies

Endometrial biopsies were collected from the uterine horn ipsilateral to the corpus luteum by using Kevorkian-Younge uterine biopsy forceps (Alcyon, Paris, France). Biopsies were cut into three pieces (sizes ≈ 4 × 4 mm^2^) and were handled as described [33]. Biopsies embedded in optimal cutting temperature (OCT) compound (VWR, Radnor, PA, USA) were 8 μm sectioned with a cryostat (Leica CM1860 Cryostat, Wetzlar, Germany) at − 20 °C under RNA-free conditions.

### Laser capture microdissection (LCM) and RNA isolation

All procedures used were as previously reported [33, 81]. The LCM process was performed by using an ArcturusXT™ Laser Capture Microdissection System and software (Applied Biosystems®, Arcturus, ThermoFisher Scietific, Waltham, MA, USA), within 1 h to avoid RNA degradation. Luminal epithelial cells (LE), glandular epithelial cells (GE) and stromal cells (ST) were harvested in sufficient numbers to obtain at least 5 ng of total RNA for each endometrial cell type. Total RNA from LCM samples was isolated and mRNA purified using the PicoPure™RNA isolation kit (KIT0202, Arcturus) following the manufacturer’s protocol. RNA integrity value (RIN values) and quantity were evaluated using the Pico RNA chip on the Agilent 2100 Bioanalyzer (Agilent technologies, Santa Clara, CA, USA). Mean RNA integrity (RIN) values obtained from LCM samples and from the full tissue samples issued from the same biopsy were similar (paired T-test; Table S2).

### RNA sequencing and data analysis

RNA sequencing libraries prepared from 24 samples (number of samples in each NEB group and endometrial cell types presented in Table S2) were prepared and sequenced on GenomEast Platform (IGBMC, Cedex, France; http://genomeast.igbmc.fr/). Libraries were built using the Clontech SMART-Seq v4 Ultra Low Input RNA kit for Sequencing as described previously [33]. Sequencing was performed on an Illumina HiSeq 4000 with 50 bp paired-end reads. Image analysis and base calling were performed using RTA 2.7.3 and bcl2fastq 2.17.1.14. All steps of the analysis were carried out as described in a sister paper [33]. Gene level exploratory analysis and differential expression were performed using the RNAseq workflow described by [82] and the update version https://www.bioconductor.org/help/course-materials/2017/CSAMA/labs/2-tuesday/lab-03-rnaseq/rnaseqGene_CSAMA2017.html. The Salmon method [83] was used to quantify transcript abundance [83]. After quantifying RNA-seq data, tximport method [84] (R package version 1.8.0) was used to import Salmon’s transcript-level quantifications to the downstream DESeq2 package (R package, version 1.20.0) for analysis of differential expressed genes (DEGs) with the statistical method proposed [85]. Principal component analysis was performed with DESeq2 and with FactoMineR (R package, version 1.4.1) using the variance stabilizing transformation output files from DESeq2. Heatmap was generated in R software using the pheatmap package (version 1.0.12) and Venn diagrams were plotted with VennDiagram package (1.6.20). DEGs of specific-endometrial cell samples were identified in comparison between SNEB and MNEB group with an adjusted *p*-value of 0.05. Volcano plot was applied to gene lists of each endometrial cell type considering the log2 fold change between SNEB and MNEB on the *x* axis and the negative log10 of the adjusted *p*-value on the *y* axis.

### Gene ontology and KEGG pathway analysis

Lists of over- or under-expressed DEGs between SNEB and MNEB were annotated into three categories of Gene Ontology (GO) pathways such as biological process (BP), cellular component (CC) and molecular function (MP) using PANTHER classification system (Protein Analysis THrough Evolutionary Relationships version 14.0, http://pantherdb.org).. Moreover, the analysis of enriched Kyoto Encyclopedia of Genes and Genomes (KEGG) pathways was performed. If a KEGG pathway was determined to be significantly enriched (Benjamini- adjusted *p*-value < 0.05), this significant process/pathway was reported. By using DEGs which are involved in significant KEGG pathways, a molecular interaction network analysis was generated by using STRING database (STRING version 11) [86] at medium confidence level (0.4) for giving an overview of the genes networks and their interactions.

#### Statistical analysis

All statistical analyses on BCS and NEFA concentrations were performed with SAS® software version 9.3 [26], using the MIXED procedure for linear mixed models. A repeated effect of time (week ante−/post-partum) within animals was tested. The correlations between test days were accounted for by specifying a correlation structure (spatial power) among residuals to consider that time intervals between samplings were not exactly the same. Backward elimination was used to build the models, excluding non-significant (*p* > 0.20) effects. The residuals from the observations generated from the mixed models were tested for normal distribution using PROC UNIVARIATE in SAS 9.3. Data deviating from a normal distribution were log-transformed. However, to improve clarity the respective log-transformed values are referred to as BCS and NEFA throughout this paper. Sampling times were treated as actual days postpartum in the analysis of the material and presentation of the results. The model used was:

$$ \mathrm{Yijk}=\mu +\mathrm{NEB}\ \mathrm{GROUP}\ \mathrm{i}+\mathrm{TIME}\ \mathrm{j}+\mathrm{NEB}\ \mathrm{GROUP}\ \mathrm{i}\ast \mathrm{TIME}\ \mathrm{j}+\mathrm{eijk} $$

If non-significant, interaction was removed from the model. Least square means (Lsmeans, ± standard error of the mean, sem) estimated by the models for the two groups of NEB were compared. A contrast option (ESTIMATE and CONTRAST statements under SAS9.3) was used to investigate differences between different combinations of time intervals (Scheffe adjustment for multiple-post ANOVA comparisons).

The results of BCS, NEFA’s concentration, and milk progesterone concentration are presented as LSmeans ± S.E.M. Differences with associated *p*-value < 0.05 were considered to be significant. In the statistical analysis of transcriptome profiles, generalized linear model was fitted and Wald test were performed to determine which of the observed fold changes were significantly different between severe and mild negative energy balance groups. *p-*values < 0.05 were considered to identify DEGs according to procedures described by [82].

## Supplementary Information

**Additional file 1: Table S1.** Lists of differentially expressed genes between SNEB and MNEB.

**Additional file 2: Table S2.** Number of samples of each cell type from MNEB and SNEB group. RNA Integrity Number (RIN)] [mean value (± s.e.m)] and average number of tissue sections required to obtain at least 10 ng of total RNA in each endometrial cell type.

**Additional file 3: Figure S1.** Experimental design including 12 cows. From energy balance profiles 9 cows were selected for LCM of endometrial tissue biopsies (5 mild NEB and 4 severe NEB cows). An arrow with dash line indicate a timing for BCS measurement and blood sampling for NEFA measurement.

## Data Availability

The data discussed in this publication have been deposited in NCBI’s Gene Expression Omnibus [87] and are accessible through GEO Series accession number GSE169638 (https://www.ncbi.nlm.nih.gov/geo/query/acc.cgi?acc=GSE169638). The gene accession numbers indicated in the additional files refer to the corresponding Ensembl identifier (https://www.ensembl.org/Bos_taurus/)(Bos taurus: ARS-UCD1.2). Gene names were retrieved using the Ensembl BioMart tool.

## Abbreviations

BCS: Body condition score
CIDR: Controlled internal drug release
DAVID: Database for annotation, visualization and integrated discovery
DEG: Differentially expressed gene
EB: Energy balance
ECM: Energy-corrected milk
Elisa: Enzyme-linked immunosorbent assay
FDR: False discovery rate
GE: Glandular epithelial cell
GO: Gene ontology
KEGG: Kyoto encyclopedia of genes and genomes
LCM: Laser capture microdissection
LE: Luminal epithelial cell
MNEB: Mild energy balance
NEB: Negative energy balance
NEFAs: Non-esterified fatty acids
NorFor: Nordic feed evaluation system
OCT: Optimal cutting temperature coumpound
PANTHER: Protein analysis through evolutionary relationships
PCA: Principal component analysis
PGE2: Prostaglandin-E2
PGF2α: Prostaglandin-F2α
REVIGO: Reduce, visualize gene ontology
RFI: Residual feed intake
RNA: Ribonucleic acid
RNA-Seq: RNA sequencing
SRB: Swedish Red breed
ST: Stromal cell
STRING: Search tool for the retrieval of interacting genes/proteins
TPM: Transcripts per million
