# Supplementary material for: Analysis of the transcriptome of bovine endometrial cells isolated by laser micro-dissection (2): impacts of post-partum negative energy balance on stromal, glandular and luminal epithelial cells
Source: BMC Genomics. 2021 Jun 18;22:450. doi: 10.1186/s12864-021-07713-z (PMC8212477; doi:10.1186/s12864-021-07713-z)
Supplement: Supplementary file 2 — Additional file 2: Table S2. Number of samples of each cell type from MNEB and SNEB group. RNA Integrity Number (RIN)] [mean value (± s.e.m)] and average number of tissue sections required to obtain at least 10 ng of total RNA in each endometrial cell type. [file 12864_2021_7713_MOESM2_ESM.docx]

| endometrial cell types | number of samples for RNA-seq | | RIN | number of tissue sections  (for 10 ng of total RNA) |
| --- | --- | --- | --- | --- |
|  | MNEB | SNEB |  |  |
| Full tissue section | - | - | 7.39 ± 0.13 | 1 ± 0 |
| Stromal cell | 5 | 4 | 7.23 ± 0.13 | 9 ± 3 |
| Glandular epithelial cell | 5 | 4 | 7.44 ± 0.13 | 13 ± 3 |
| Luminal epithelial cell | 5 | 1 | 7.75 ± 0.15 | 35 ± 3 |

Table S2: Number of samples of each cell type from MNEB and SNEB group. RNA Integrity Number (RIN)] [mean value (± s.e.m)] and average number of tissue sections required to obtain at least 10 ng of total RNA in each endometrial cell type.
