# Supplementary figures and images for: Analysis of the transcriptome of bovine endometrial cells isolated by laser micro-dissection (2): impacts of post-partum negative energy balance on stromal, glandular and luminal epithelial cells
Source: BMC Genomics. 2021 Jun 18;22:450. doi: 10.1186/s12864-021-07713-z (PMC8212477; doi:10.1186/s12864-021-07713-z)

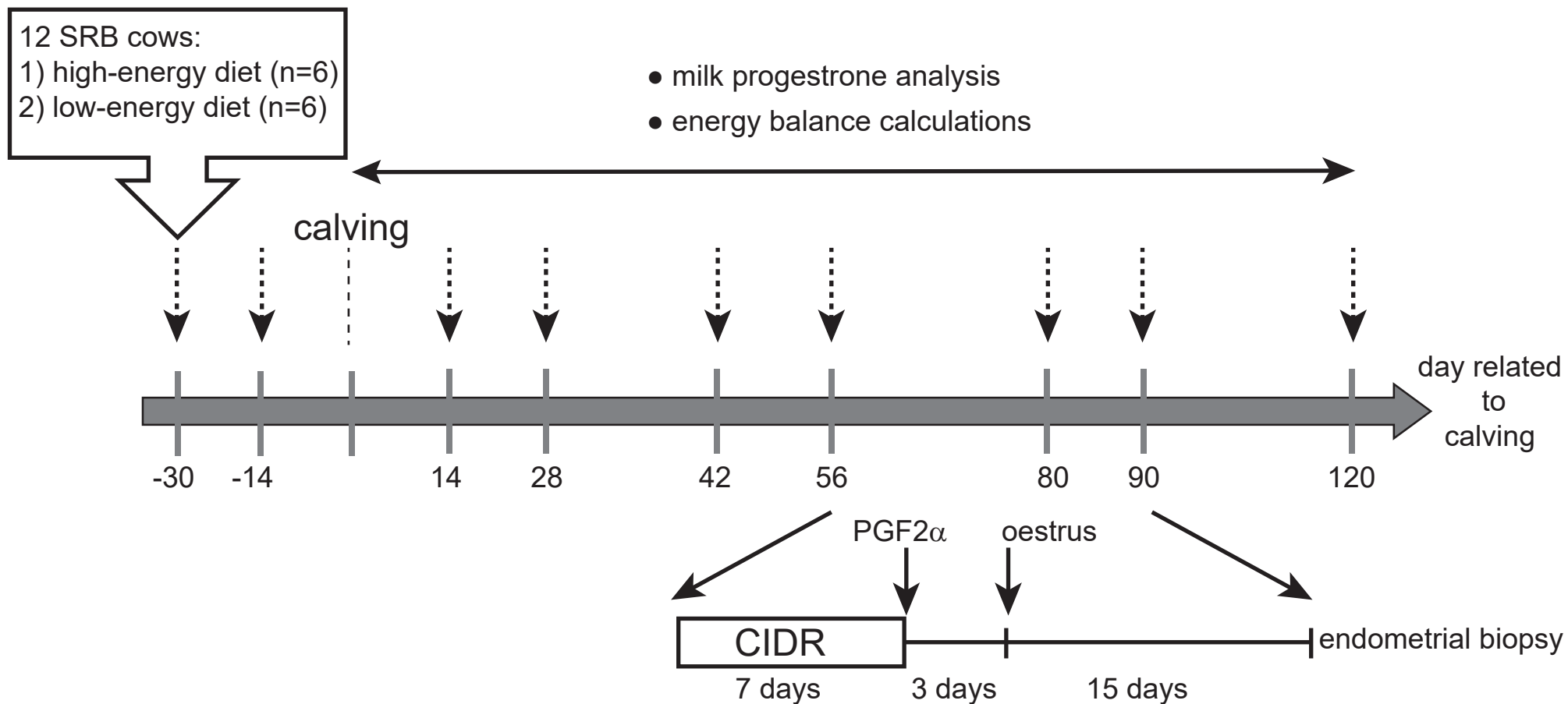

figure S1

Supplement: Supplementary file 3 — Additional file 3: Figure S1. Experimental design including 12 cows. From energy balance profiles 9 cows were selected for LCM of endometrial tissue biopsies (5 mild NEB and 4 severe NEB cows). An arrow with dash line indicate a timing for BCS measurement and blood sampling for NEFA measurement. [file 12864_2021_7713_MOESM3_ESM.pdf]
